# A Comparison of Acute Neurocognitive and Psychotomimetic Effects of a Synthetic Cannabinoid and Natural Cannabis at Psychotropic Dose Equivalence

**DOI:** 10.3389/fpsyt.2022.891811

**Published:** 2022-05-19

**Authors:** Eef Lien Theunissen, Kim Paula Colette Kuypers, Natasha Leigh Mason, Johannes Gerardus Ramaekers

**Affiliations:** Department of Neuropsychology and Psychopharmacology, Faculty of Psychology and Neuroscience, Maastricht University, Maastricht, Netherlands

**Keywords:** cannabinoids, THC, JWH-018, psychotropic dose equivalence, neurocognitive effects, psychotomimetic effects

## Abstract

Due to differences in potency, efficacy, and affinity for CB1 receptors, similarities and differences in psychoactive effect profiles of natural cannabis and synthetic cannabinoids (SCs) cannot reliably be derived from equipotent dose comparisons. Instead, the current study proposes to compare the intrinsic psychoactive effects of natural cannabis (THC) and an SC, JWH-018, at psychotropic dose equivalence. Participants from two placebo-controlled studies were matched for their levels of subjective high to compare neurocognitive and psychotomimetic effects of THC and JWH-018. At equal subjective intoxication levels, both drugs impaired psychomotor, divided attention, and impulse control, with no significant difference between the two drugs. Both drugs also caused significant psychotomimetic effects, but dissociative effects were considerably more pronounced for JWH-018 than THC. We conclude that psychotropic dose equivalence provides a uniform approach for comparing the neurocognitive and psychotomimetic profiles of CB1 agonists, which can also be applied to other drug classes.

## Introduction

The availability of novel psychoactive substances (NPS), which mimic the effects of traditional drugs of abuse, has increased rapidly over the past decades (1). NPS include diverse classes of substances, such as synthetic cannabinoids, synthetic cathinones, phenethylamines, piperazines, tryptamines, aminoindanes, and NPS opioids (2). Although NPS use is less common than the use of other illicit drugs (3), the harm caused to individuals can be quite serious, ranging from acute impairment and adverse effects, to drug addiction (1).

One class of NPS, synthetic cannabinoids (SCs), includes compounds initially designed since the 1970's by researchers to investigate the cannabinoid system and to explore new therapeutic indications, e.g., pain disorders and cancer (4, 5). However, in the early 2000s, these compounds (e.g., JWH-018) appeared in smoking mixtures, such as Spice or K2, which were advertised as “natural” alternatives for cannabis. These smoking mixtures became popular rapidly, especially in countries where recreational use of cannabis was illegal or in situations where users wanted to avoid detection in routine drug screening (6). However, it also became clear from anecdotal reports that these SCs come with serious side effects, such as agitation, psychotomimetic effects, and cardiac events (7–10), as well as fatalities (11, 12). As a result, more and more of these SCs were added to the list of controlled substances in progressively more countries. In response to these bans, the content of the smoking mixtures has changed continuously, with new and more potent SCs being released on the market at an increasing speed (13–15).

SCs bind to the central cannabinoid receptors (CB1 and CB2), which are also the target receptors for Δ9-tetrahydrocannabinol (THC), the main psychoactive component of cannabis. However, the effects elicited by most SCs are more powerful than the effects of natural cannabis (16). The risk of emergency medical treatment is about 30 times greater following the use of SCs than following cannabis (17), with tachycardia, agitation, and nausea as the most frequently reported adverse events (8). Cases of SC intoxication often display cognitive impairment (16). Compared to natural cannabis users and non-cannabis users, frequent SC users were found to perform worse on cognitive tasks, including working memory, inhibition, and long-term memory (18). The most well-known adverse effects linked to SCs are probably psychological symptoms, including agitation, anxiety, and psychosis (16, 19). Psychotomimetic and dissociative effects, often referred to as “zombie effect,” have been noted in up to 28% of the people who admitted using an SC (20, 21). Although the symptoms of SCs overlap with those of cannabis, the stronger and more unpredictable effects of SCs cause the majority of users to express a preference for natural cannabis (22).

Most SCs have a specifically high affinity for CB1 (16, 23–26). Furthermore, they act as agonists with high efficacy (i.e., act as a full agonist) (23, 24, 27). This is in contrast to THC, which has low efficacy, low affinity for CB1, and is less potent, and thus is not able to stimulate cannabinoid receptors to the same degree as SCs (24, 28). Consequently, the behavioral effects of a given dose of an SC are not simply comparable to those of an equivalent dose of THC. In pioneering studies conducted in our lab with an early SC, JWH-018, we demonstrated that a low dose produced significant psychomotor and cognitive impairment, as well as psychotomimetic symptoms (29–32) even at subjective intoxication levels that were lower than what is normally reported for natural cannabis. It seems therefore appropriate to take the level of subjective intoxication into account when establishing the psychotropic dose equivalence between an SC and natural cannabis.

In the present manuscript, we defined psychotropic dose equivalence as the dose at which an identical level of subjective high is achieved with an SC and natural cannabis. Subsequently, we aimed to compare neurocognitive and psychomimetic effects of an SC (i.e., JWH-018) and cannabis in participants from two previous placebo controlled studies (31–34) that were matched for their levels of subjective high. We expected that a comparison at psychotropic dose equivalence would allow for an objective comparison of the neurocognitive and psychotomimetic profiles of JWH-018 and THC.

## Materials and Methods

**Figure 1:**
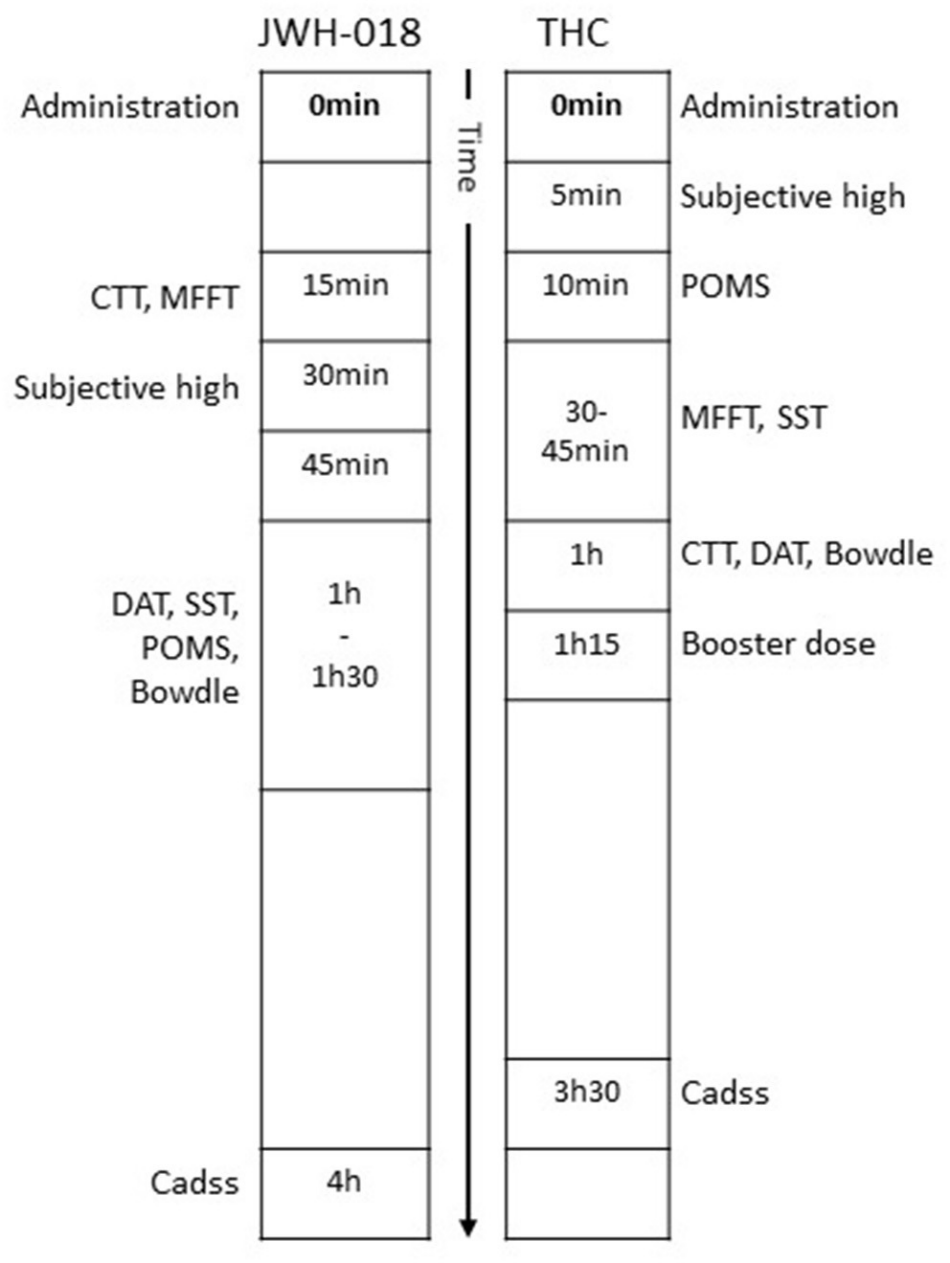
Timeline of subjective questionnaires and cognitive tests relative to time of drug administration.

Subjective and performance data analyzed in this study comes from two studies with a comparable design and setup (31–34). Both studies were placebo-controlled, cross-over studies examining the acute effect of either cannabis (THC) or JWH-018 in healthy cannabis users. The type of cannabis users was comparable in that both studies included occasional users (i.e., cannabis use between 8 and 120 times/year). The studies used the same test battery and questionnaires to measure cognitive and subjective effects that were administered within a similar time window relative to drug dosing. A summary of the subjective and objective tests applied in both studies, and at which time after dosing they were administered, is provided in Figure 1.

### Samples and Matching

#### THC Study

THC data was taken from a previous study from our group (33, 34). In this randomized, double-blind experimental study, 122 cannabis-experienced (at least 8 times/year) participants received 450 μg/kg THC (cannabis plant material, divided into two doses of 300 and 150 μg/kg; i.e., 21 and 11 mg for a 70 kg person), cocaine, and placebo on three separate test days. Cannabis treatment was split into two doses, with the second one given 1 h after the first dose. Cannabis was prepared from batches containing 11–12% THC, while placebo cannabis consisted of Knaster Hemp. Cannabis and placebo were administered using a vaporizer (Volcano) which heated the materials to a temperature of about 225 °C, while storing the vapor in a polythene bag equipped with a valved mouthpiece. Subjects were instructed to inhale according to a standardized manner, inhaling deeply and holding their breath for 10 s after each inhalation. The bag had to be emptied completely, taking about 2–3 min. Subjective and performance measures were taken within 3.5 h after the first administration (see Figure 1). The cocaine condition has been omitted from the current study.

#### JWH-018 Study

Subjective and performance data after an acute dose of JWH-018 was taken from a previous study performed by our group (31, 32). In this placebo-controlled, cross-over study, 24 healthy occasional cannabis users inhaled the vapor of 75 μg JWH-018/kg body weight and placebo on two separate test days (i.e., 5.25 mg for a 70 kg person). Placebo consisted of Knaster Hemp (Zentauri, Germany), a herbal blend with hemp aroma (0% THC). Both treatments were administered *via* a vaporizer pen, which heated the materials to ~380°C. Participants inhaled the vapor in five intakes, according to a strict inhalation regimen. A booster dose of 50 μg JWH-018/kg body weight was administered in case participants did not show a subjective response (i.e., a minimum subjective high score of 30% was required) within 15 min after administration of JWH-018. Performance and subjective measures were taken in the 4 h following drug administration (or booster in case needed) (Figure 1). Subjective intoxication was measured regularly after JWH-018 treatment but was maximal at 30 min post drug.

#### Subjective Intoxication

Subjective high was self-rated on a 10 cm Visual Analog Scale (VAS), with 0 indicating “not high at all,” and 10 indicating “extremely high.” Participants reported their highest scores within the first hour after administration for both THC and JWH-018, followed by a return to baseline levels in the following hours (32, 35, 36). In the THC study, subjective high score was determined 5 min after the first administration, when subjective intoxication for THC was maximal in occasional users (36). In the JWH-018 study, subjective intoxication was maximal at 30 min post drug.

#### Matching

To ensure comparable cannabis use patterns across the samples, only participants who used cannabis a maximum of 10 times/month were included from the THC and JWH-018 study. Subsequently, participants with a comparable subjective high score after THC or JWH-018 were selected (max, mean, SD difference was 0.8, 0.28, and 0.36). This resulted in 24 participants of the THC sample who could be matched to the participants in the JWH-018 sample. The characteristics of both samples are shown in Table 1.

**Table 1:** Demographics of the participants in the THC and JWH-018 sample.

	**THC (*N* = 24)**	**JWH-018 (*N* = 24)**
Male/female	20/4	10/14
	***Mean (SD)***	***Mean (SD)***
Age (years)	21.95 (4.0)	22.8 (3.05)
Subjective high score (cm)	6.46 (2.0)	6.41 (1.9)
Estimated cannabis use (times) in the month	5.5 (2.5)	3.4 (2.3)

### Performance Measures

#### Critical Tracking Task

The CTT is a psychomotor test that assesses the participant's ability to control a displayed error signal in a first-order compensatory tracking task (37). Error is displayed as a horizontal deviation of a cursor from the midpoint on a horizontal, linear scale. Compensatory joystick movements null the error by returning the cursor to the midpoint. Total duration of the task is ~3 min. The frequency at which the participant loses control is the critical frequency or lambda-c (λ_c_). The test included five trials, of which the lowest and the highest score were removed; the average of the remaining scores is taken as the final CTT-score. This test has repeatedly been shown to be sensitive to the effects of many sedative drugs, including cannabis (36, 38–40).

#### Divided Attention Task

The DAT measures the ability to divide attention between two tasks performed simultaneously (41). Participants have to perform the same tracking task as described above, but now at a constant difficulty level. As a secondary task, the subject monitors 24 single digits presented in the corners of the computer screen. The participants have to react to the target number “2” by removing their foot as fast as possible from a pedal switch. Duration of the task is 12 min. The mean absolute tracking error (in mm) and the number of control losses are the performance measures of the primary task. The number of misses, false alarms, and mean reaction time (msec) of the responses to the target number, are the performance measures in the secondary task. Performance in this test has proven to be sensitive to the effects of many sedative drugs (36, 38–40).

#### Stop Signal Task

The SST measures motor impulsivity, which is the inability to inhibit an activated or pre-cued response leading to errors of commission. The current test is adapted from an earlier version (42) and has been validated for stimulant and sedative drug effects (43). The task requires participants to make quick responses to visual go-signals and to inhibit their response if a subsequent visual stop-signal, i.e., “^*^”, appears in one of the four corners of the screen. Total task duration is ~8 min. Dependent variables are go reaction time (ms), stop reaction time, number of correct responses, omission (not responding on go-trials), and commission errors (not inhibiting a response to a no go trial). Stop reaction time represents the estimated mean time required to inhibit a response. Stop reaction time is calculated by subtracting the stop signal delay from the reaction time on go-trials associated with *n*^th^ percentile of the reaction time (RT) distribution (44).

#### Matching Familiar Figures Test

The MFFT measures reflection impulsivity, which is the tendency to reflect on the validity of problem-solving under the particular condition of several possible alternatives. The test involves simultaneous presentation of a target figure positioned on the left of the screen and an array of six alternatives on the right half of the screen, all except one differing in one or more details from the target figure. The participants are asked to select from the alternatives the figure that exactly matches the target figure as quickly as possible. Task duration is ~5 min. Two dependent measures, mean latency to first response (ms) and the total number of errors, are automatically recorded. In addition, an impulsivity score (I-score) is calculated by subtracting the standard score of the mean latency to the first response from the standard score of the total number of errors committed. An efficiency score (E-score) is calculated by summing the standard score of the mean latency to the first response with the standard score of the total number of errors committed.

### Subjective Measures

#### Clinician Administered Dissociative States Scale

The Clinician Administered Dissociative States Scale (CADSS) (45) comprises of 19 self-rated items, ranging from 0 “not at all” to 4 “extremely.” It is divided into three components: *depersonalisation* (5 items), *derealisation* (12 items), and *amnesia* (2 items). A total dissociative score is achieved by summing all items. The CADSS is designed to be a standardized measure of present-state dissociative symptomatology and was previously found to be sensitive to dissociative effects of psychedelics and drugs of abuse, such as ketamine and THC (46–48).

#### Bowdle Visual Analog Scales

Psychedelic symptoms are assessed using a 13-item VAS (49). Two scales measure subjective “*high*” and “*drowsiness*.” From the other scales, composite scores of “*internal perception*” (reflecting inner feelings that do not correspond with reality) and “*external perception*” (reflecting a misperception of an external stimulus or a change in the awareness of the subject's surroundings) are calculated (50).

#### Profile of Moods States

The POMS is a self-assessment mood questionnaire with 72 items, rated on a 5-point Likert scale, with 0 being “not at all” to 4 “extremely.” Participants have to indicate to what extent these items were representative of their mood at that moment. Eight mood states are classified and quantified by calculating the sum score of associated items for each mood state, i.e., *anxiety, depression, anger, vigor, fatigue, confusion, friendliness*, and *elation*. Two composite scales are derived; *arousal* and *positive* mood (51).

### Procedure

Participants for both studies were recruited *via* advertisements. Data collection in the THC study was part of a more extensive study that included fMRI scanning after the booster dose. Participants of both studies were only included if they had prior experience with cannabis. They were medically examined by a physician, who checked for general health and took blood and urine samples for standard chemistry and hematology. Both studies were approved by the standing Medical Ethics Committee of Maastricht University and were carried out in compliance with the revision of the Declaration of Helsinki applicable at that time (i.e., Seoul, 2008; Fortaleza, 2013) and the International Conference on Harmonization guidelines for Good Clinical Practice. A permit for obtaining, storing, and administering cannabis and JWH-018 was obtained from the Dutch drug enforcement administration. All participants gave written informed consent and received financial compensation for their participation. All tests were taken after acute administration of the drug (see Figure 1).

### Statistical Analyses

A student's *t*-test was used to compare differences in monthly cannabis use, while chi-square test was used to compare distributions of sexes between the two studies.

Outcome data that was normally distributed was analyzed using GLM Repeated Measures ANOVA, with Drug (placebo and drug condition (i.e., THC or JWH-018) as within-subject factor and Study (THC and JWH-018 study) as between-subject factor. Partial eta squared (partial η^2^) is reported to demonstrate the effect's magnitude and is based on Cohen's f, which defines small, medium, and large effect sizes as, respectively 0.10, 0.25, and 0.40, which corresponds to partial η^2^ values of 0.01, 0.06, and 0.14 (52). Subsequently, difference scores (THC/JWH-018 minus placebo) were analyzed with pairwise comparisons with a *t*-test for independent samples.

Non-normal distributed data was analyzed with the non-parametric Wilcoxon signed-rank test, to test for significant differences between the drug condition (THC or JWH-018) and placebo. Difference scores (THC or JWH-018–placebo) were tested with a Mann-Whitney test, to assess for differences between THC and JWH-018. A *p*-value of <0.05 was considered statistically significant. The effect size (*r*) for non-parametric tests is calculated by dividing the Z-score by the square root of N, indicating a large effect size using Cohen's d criteria of 0.10, 0.30, and 0.50 for defining small, medium, and large effect sizes. All statistical tests were conducted using IBM SPSS statistics, version 26.

### Missing Data

There were several missing data due to technical malfunctioning or the participant not being capable of performing the test. For the THC study, CTT data from one participant in the THC condition was missing, as was DAT data from three participants in the placebo and three other participants in the THC condition. In the JWH-018 study, the following was missing: CTT-score for one participant in the JWH-018 condition; DAT for one participant in the placebo and one participant in the JWH-018 condition; SST data for one participant in the JWH-018 condition, and MFFT data for one participant in the placebo and one participant in the JWH-018 condition. Missing data of these participants were replaced by the study and condition's average before entering the RM ANOVA analysis.

## Results

### Demographics

Participants in both groups used cannabis <10 times/month, nevertheless, the average consumption in the groups was statistically different [*t*_(46)_ = 3.17, *p* < 0.01]. Also, the division of sexes was significantly different according to a Chi-square test [*X* ^2^(1, *N* = 48) = 8.89, *p* < 0.01].

### Subjective High

**Figure 2:**
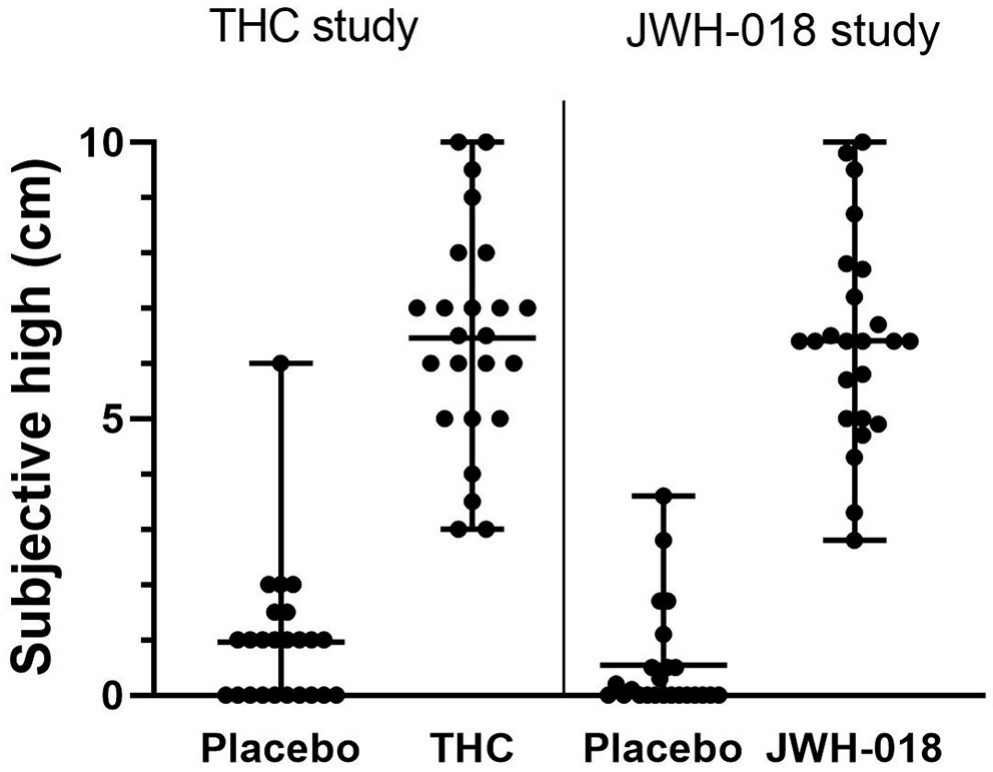
Scatter plot with mean and range of subjective high scores in placebo and drug conditions in both samples.

Individual subjective high scores per treatment condition are presented in Figure 2. Mean subjective high score was 6.46 after THC and 6.41 after JWH-018 administration. *Subjective high* score demonstrated a significant effect of Drug [*F*_(1, 46)_ = 329.1; *p* < 0.001, $\eta_{p}^{2}$ = 0.88]. No significant effect of Study or Drug x Study was found, confirming that subjective high in both samples was comparable.

### Cognitive and Psychomotor Tests

#### Critical Tracking Task

**Figure 3:**
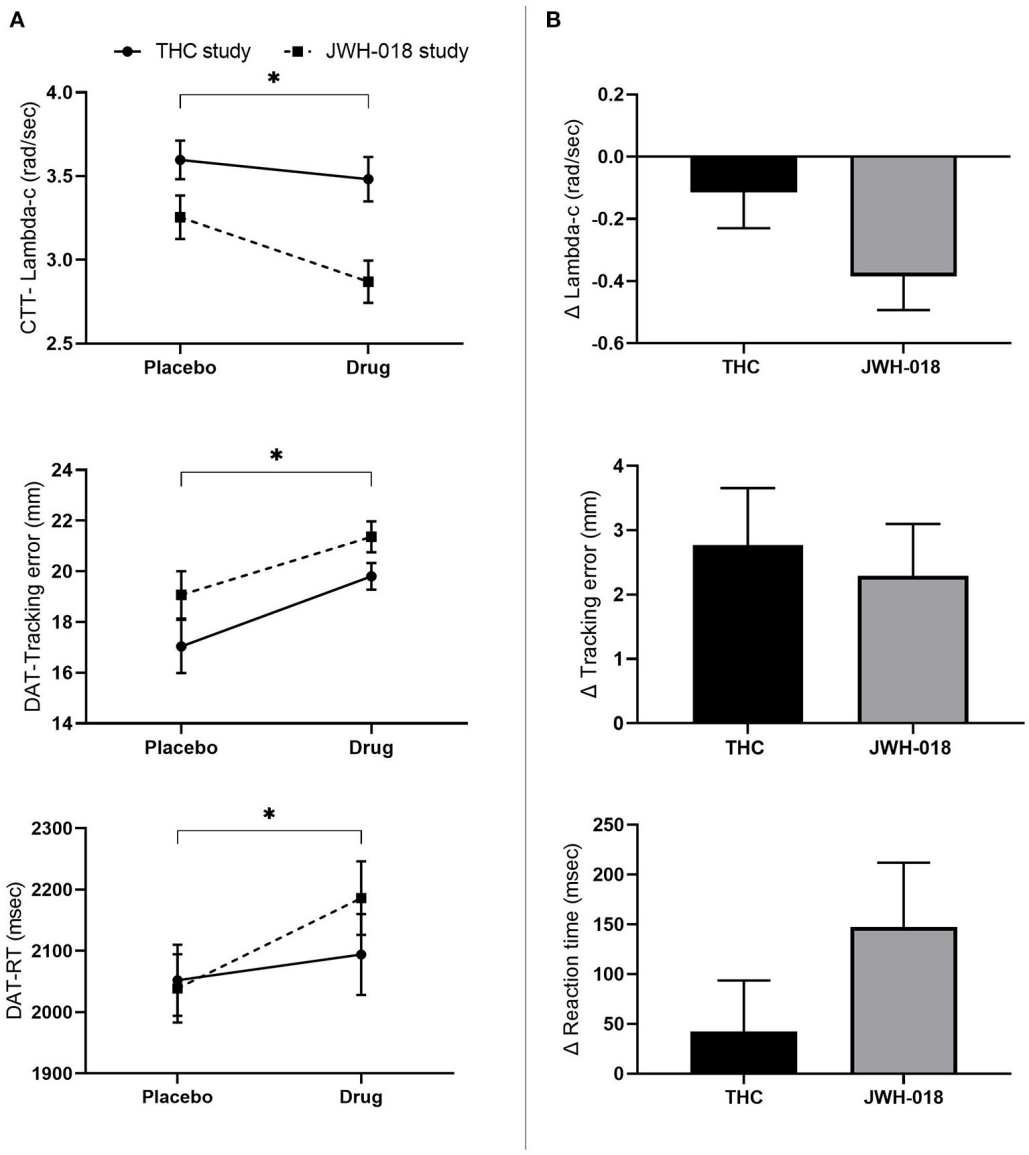
**(A)** Mean (SEM) values for lambda-c in the critical tracking task, and tracking error and reaction time in the divided attention task for placebo and drug conditions, in the THC and JWH-018 study. **(B)** Lambda-c in the critical tracking task, and Tracking error and reaction time in the divided attention task for both drug conditions shown as difference scores (relative to placebo). ^*^Significant difference between the active condition (THC/JWH-018) and placebo.

GLM Repeated measures ANOVA showed a significant effect of Drug and Study on *CTT score* [*F*_(1, 46)_ = 9.8; *p* = 0.003, $\eta_{p}^{2}$ = 0.18; *F*_(1, 46)_ = 8.88; *p* = 0.005, $\eta_{p}^{2}$ = 0.16], indicating that drug conditions impaired performance (Figure 3A), as compared to placebo. Subsequent *t*-test analysis on the difference scores showed no significant difference between the change in CTT scores caused by THC or JWH-018 (Figure 3B).

#### Divided Attention Task

GLM Repeated measures ANOVA showed a significant effect of Drug on *tracking error* [*F*_(1, 46)_ = 17.66; *p* < 0.01, $\eta_{p}^{2}$ = 0.27] and *RT* [*F*_(1, 46)_ = 5.25; *p* = 0.027, $\eta_{p}^{2}$ = 0.10]. *Tracking error* and *RT* increased after the drug conditions (Figure 3A). *T*-test analysis showed that the change in tracking error and RT did not differ significantly between THC and JWH-018 (Figure 3B).

*Control losses* and *false alarms* were analyzed with the non-parametric Wilcoxon signed-rank test, which showed that these were significantly higher after drug conditions compared to the placebo conditions (*Z* = −4.83; *p* < 0.01; *r* = 0.49 and *Z* = −4.14; *p* < 0.01; *r* = −0.42). The change in number of control losses and false alarms was not different between THC and JWH-018, according to Mann-Whitney tests.

#### Stop Signal Task

**Figure 4:**
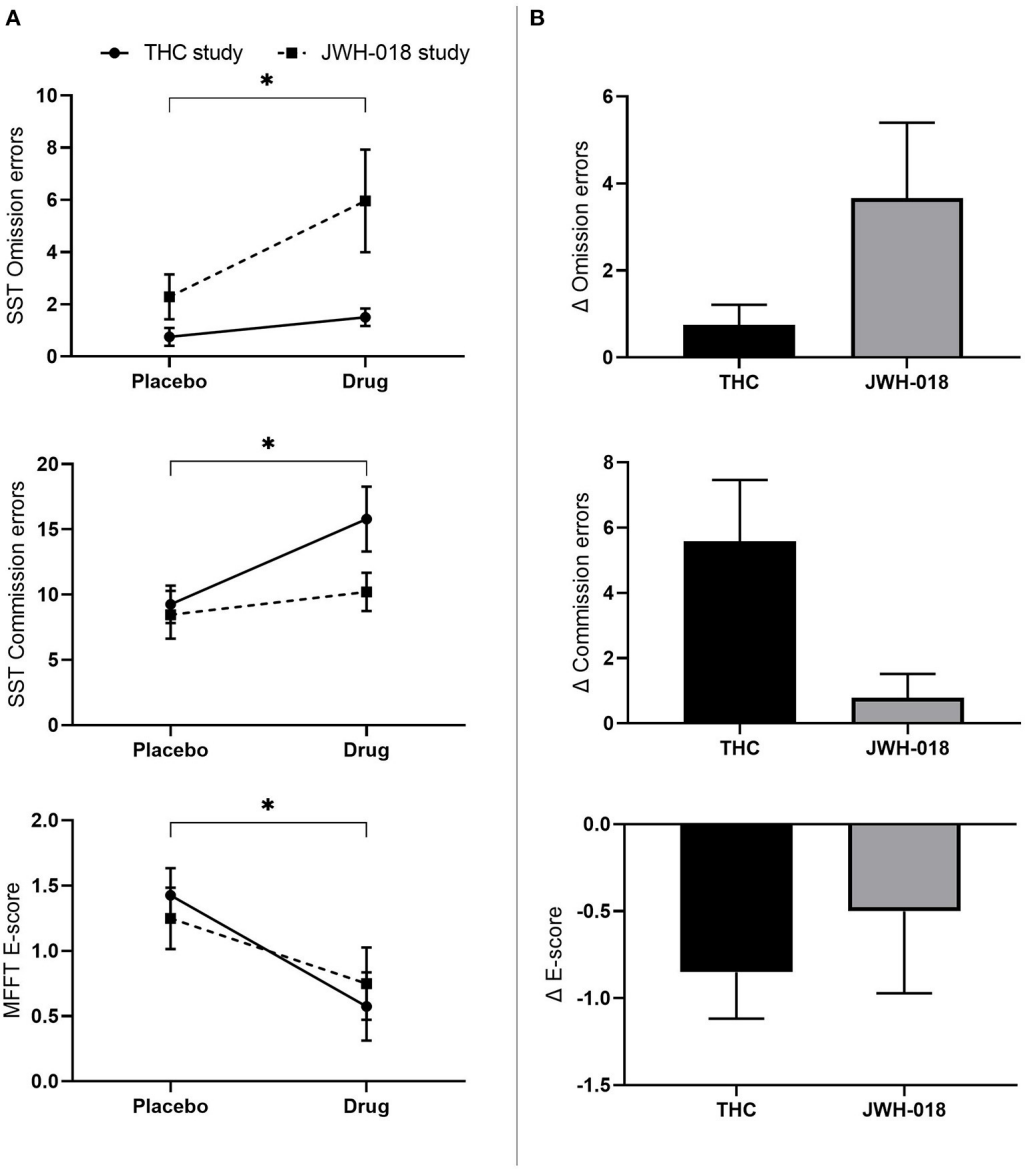
**(A)** Mean (SEM) number of omission and commission errors in the stop signal task and E-score in the matching familiar figures test for placebo and drug conditions, in the THC and JWH-018 study. ^*^Significant difference between the active condition (THC/JWH-018) and placebo. **(B)** E-score in the matching familiar figures test for both drug conditions shown as difference scores (relative to placebo).

Non-parametric testing showed that the number of *omission and commission errors* increased (*Z* = −2.50; *p* = 0.012; *r* = −0.25 and *Z* = −2.68; *p*=0.007; *r* = −0.27) in the drug conditions compared to placebo (Figure 4A). No effect was found on Stop or Go reaction time. Paired comparison of change scores from placebo demonstrated that the changes in number of omission and commission errors did not differ significantly between THC and JWH-018 (Figure 4B).

#### Matching Familiar Figures Test

GLM Repeated measures ANOVA demonstrated a significant effect of Study on *Latency* (*F*_(1, 46)_ = 9.27; *p* = 0.004) with participants in the JWH-018 study showing slower responses. GLM did not show an effect on *I-score*.

Non-normally distributed *E-score* and *errors* were analyzed using Wilcoxon signed-rank test, and this showed that the *E-score* significantly decreased after the drug conditions compared to placebo (*Z* = −2.17; *p* = 0.03; *r* = −0.22) (Figure 4A). No significant difference was found in the number of errors. Pairwise comparison of change scores from placebo indicated no significant difference on E-scores between THC and JWH-018 (Figure 4B).

### Subjective Questionnaires

#### POMS

**Figure 5:**
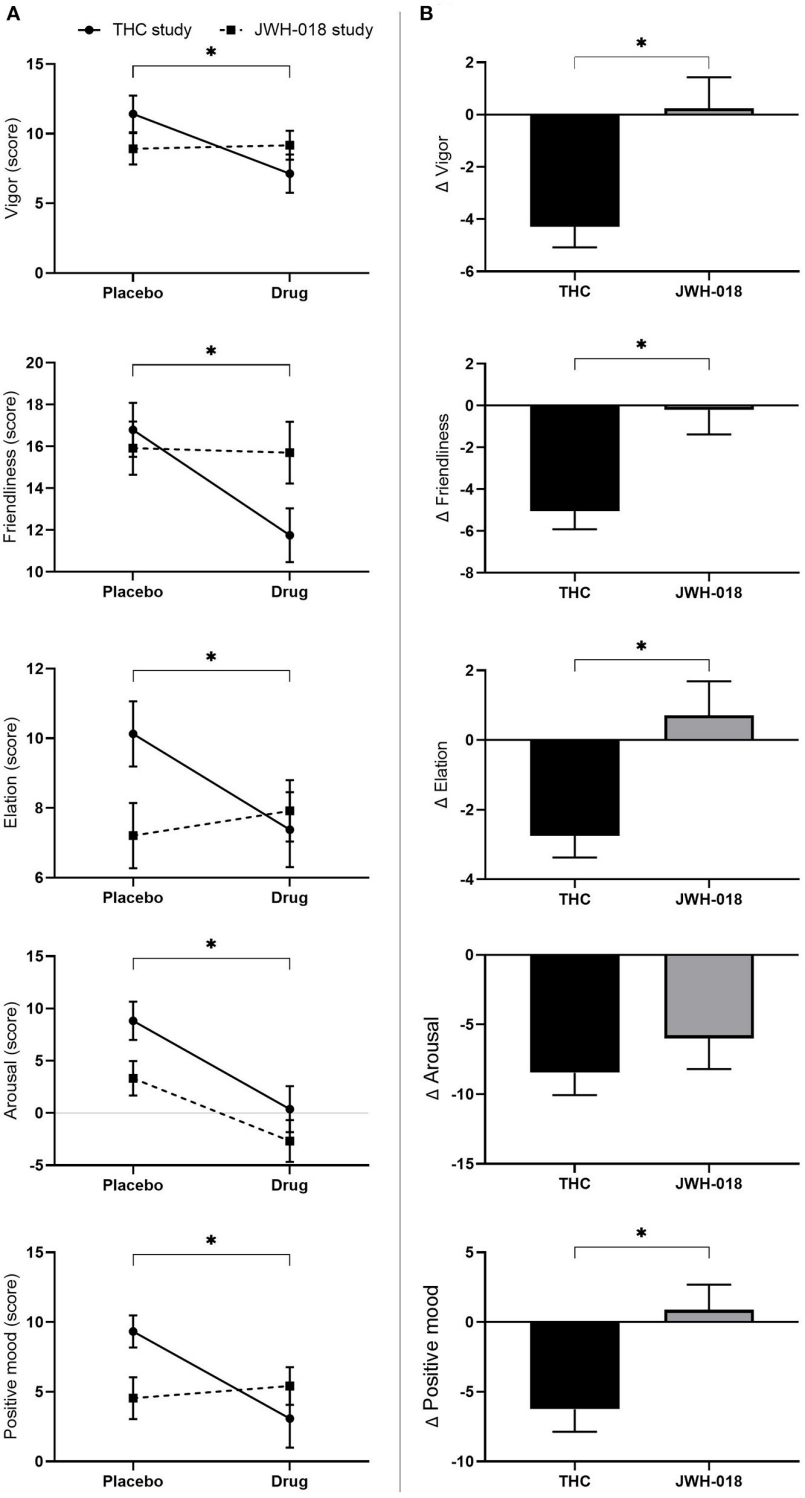
**(A)** Mean (SEM) scores on the POMS scales vigor, friendliness, elation, arousal, and positive mood, for placebo and drug conditions, in the THC and JWH-018 study. ^*^Significant difference between the active condition (THC/JWH-018) and placebo. **(B)** Scores on the POMS scales vigor, friendliness, elation, arousal, and positive mood for both drug conditions shown as difference scores (relative to placebo). ^*^Significant differences between THC and JWH-018.

All scales of the POMS were analyzed using non-parametric testing. Wilcoxon signed-rank test showed that all scales of the POMS, except anger, were significantly different in the drug conditions compared to placebo (see Supplementary Material for *Z* and *p*-values). While scores on the *anxiety, depression, fatigue, confusion* scale increased, scores on *vigor, friendliness, elation, arousal, and positive mood* decreased after treatment (Figure 5A).

Pairwise comparison showed that scores on *vigor, friendliness, elation, and positive mood* were significantly different between THC and JWH-018 (see Supplementary Material for *U* and *p*-values) (Figure 5B).

#### CADSS

**Figure 6:**
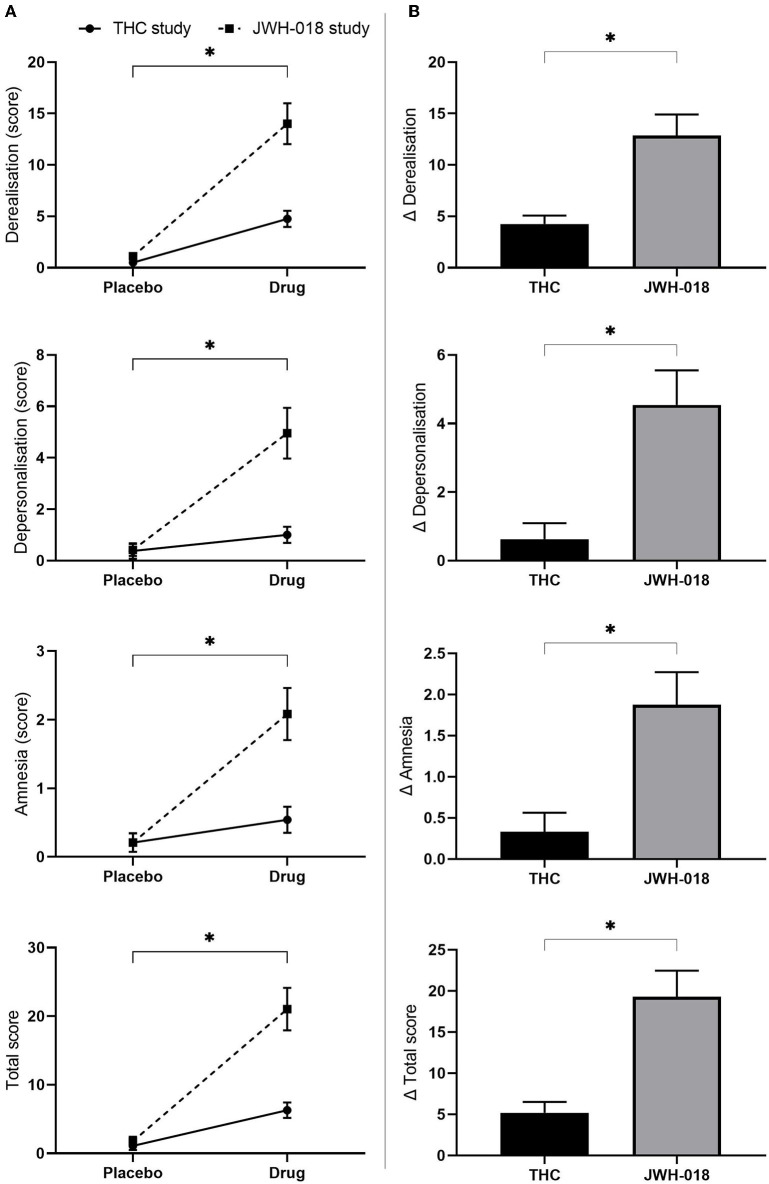
**(A)** Mean (SEM) scores on the CADSS derealisation, depersonalisation amnesia, and total score for placebo and drug conditions, in the THC and JWH-018 study. ^*^Significant difference between the drug condition (THC or JWH-018) and placebo. **(B)** scores on the CADSS derealisation, depersonalisation amnesia, and total score for both drug conditions shown as difference scores (relative to placebo); significant differences between THC and JWH-018.

All scales of the CADSS were analyzed non-parametrically, and responses were significantly higher in the drug conditions than in placebo (see Supplementary Material for *Z* and *p*-values) (Figure 6A). Pairwise comparison of change scores from placebo demonstrated that the scores in the JWH-018 condition were significantly higher than in the THC condition (see Supplementary Material for *U* and *p*-values) (Figure 6B).

#### Bowdle

Wilcoxon signed-rank tests showed that all scales of the Bowdle were significantly increased after drug treatment compared to placebo (see Supplementary Material for *Z* and *p*-values). Pairwise comparison of change scores from placebo showed that there was no difference in the scores on the Bowdle scales between THC and JWH-018.

## Discussion

Although SCs act on the same receptors as THC, it is not straightforward to compare these compounds based on equipotent doses due to the additional differences in affinity, and efficacy. Instead, in this study, the effects of an SC were compared with cannabis (THC), based on psychotropic dose equivalence as represented by subjective intoxication levels. We selected a group of occasional cannabis users who took part in a large THC study and, based on their acute subjective intoxication score, matched these to a group of participants who took part in a JWH-018 study. The design and measures used in both studies were similar, making these datasets ideal for conducting this comparative analysis.

When using equipotent doses in our earlier study [i.e., with JWH-018 being 4–5 times as potent as THC, a dose 5 times as low as a standard THC dose was selected for JWH-018 (i.e., 3 mg JWH-018)], levels of subjective intoxication produced by JWH-018 were much lower than what has been shown previously for typical THC doses (29). In the current study, using psychotropic dose equivalence, i.e., at similar levels of subjective intoxication after both cannabinoids (i.e., 6.46 and 6.41 cm), comparable effects were demonstrated on cognitive performance tests. Both drugs impaired performance on psychomotor, divided attention, and impulsivity tasks with similar magnitudes. Likewise, no significant differences between THC and JWH-018-induced levels of cognitive impairment were found. Subjective questionnaires demonstrated that both drugs caused significant psychotomimetic effects but that the dissociative effects were considerably stronger for JWH-018 than for THC, while positive mood states were more affected by THC. Psychotomimetic effects of JWH-018 at psychotropic dose equivalence in the present study were higher than those observed at equipotent dose equivalence in a previous study (30), which underscores the relevance of distinguishing between these comparative approaches.

Dissociative symptoms, known to occur in psychiatric disorders such as schizophrenia, have been reported during cannabis intoxication (53), and the link between the use of cannabis and psychosis has been reported recurrently (54–58). The risk of an adverse psychotomimetic experience after cannabis use is dose-related, with higher doses increasing the risk (59–61). The current finding that synthetic cannabinoids induce stronger psychotomimetic effects is in line with the many case reports and epidemiological data showing SCs to elicit psychotomimetic effects in vulnerable populations or patients with schizophrenia and healthy individuals (19, 55, 56, 62–64). The lack of cannabidiol (CBD), the non-psychoactive constituent of cannabis, might explain the increased risk of experiencing psychotomimetic effects after SC use. CBD, which is present in different ratios in natural cannabis, has been found to protect against THC's psychotic effect (65–69). It has also been suggested that, due to the higher efficacy, SCs alter the function of the neurotransmitter systems involved in schizophrenia, such as dopamine and glutamate, to a considerable extent (70). In addition, SCs yield metabolites with partial to full agonist activity (24), which again can interact with these neurotransmitter systems, leading to an increased effect. For THC, acute administration has been found to increase glutamate and dopamine levels (71), and increases in striatal glutamate levels were found to underlie the acute psychotomimetic effects of intravenously administered THC (72). It can be hypothesized that the association between striatal glutamate and psychotomimetic symptoms is even more prominent for SC due to their higher levels of efficacy. Studies into the effect of SCs on the neurotransmitter systems involved in schizophrenia are needed to determine the role of these systems in explaining the strong psychotomimetic effects compared to natural cannabis.

As in our previous studies (32, 34), the current analysis demonstrated that drug treatment (THC and JWH-018) affected mood states, with the negative states (except anger) increasing and the positive states decreasing. The decrease in friendliness, vigor, elation, and positive mood was apparent after THC, while both drugs decreased arousal. The effects of THC on mood have not been consistent in previous studies. Some studies showed no effect of THC on mood (73–75), while others demonstrated negative effects (76–78), whereas a more recent study showed elevated scores on positive states (79, 80). These inconsistencies in the effect of THC on mood can possibly be explained by essential differences in study designs, such as differences in dose, route of administration, time of assessment after dosing, and participants' drug use history. SCs users also often report negative mood changes (81) when intoxicated. This negative effect on mood was confirmed for JWH-018 in the current comparative analysis and in the previous studies (30, 32). However, in the current study, the changes in mood after JWH-018 appeared to be less prominent than after THC. This could be due to baseline differences in mood states, which were incorporated in the previous JWH-018 study (30, 32). However, no baseline measurement of mood was taken in the THC-study, hence no correction for baseline differences could be applied in the current study.

Participants in the current study were matched on subjective intoxication levels to accomplish psychotropic dose equivalence. However, there were significant differences in the average monthly consumption of cannabis and the males/females ratio in the two groups (83.3% males in the THC study vs. 41.7% in the JWH-018 study). With respect to cannabis use history, an earlier study comparing a large sample of infrequent and daily cannabis users demonstrated that the acute effects on neurocognitive performance were similar across users irrespective of their cannabis use history (33). With the current participants' use history falling in a much narrower range of cannabis use (i.e., excluding using more than 3 times/week), it is unlikely that this small difference in cannabis use history might have contributed to different findings for THC and JWH-018. With regard to sexes, there have been suggestions that women are more sensitive to the effects of THC than men (82). However, several studies were unable to confirm this, showing no differences between men and women in the acute effect of THC on neurocognitive function (80, 83). In the current study, it is also unlikely that sex differences significantly affected the study results. Men were overly represented in the THC study, but nevertheless, the effects of THC were apparent on a wide range of measures. Overall, by using the subjective high scores as the matching factor between our groups, potential confounding of differences in sex and cannabis use history between participants of the two studies were eliminated.

The proposed method of comparing drugs at psychotropic dose equivalence can also be applied to other drugs of abuse. Especially with the large number of NPS entering the market since the early 2000s, it has been challenging to determine the risks associated with these new compounds. The EMCDDA (84) has, nevertheless, recommended that individual health risks of NPS be assessed in pharmacological studies in humans and include psychological and behavioral measures. Moreover, the EMCDDA guidelines advise evaluating the risk of novel NPS relative to traditional drugs of abuse. However, when comparing different compounds within various drug classes, different neurotransmitter binding profiles are typical. Psychedelic drugs, for example, all act as agonists at the serotonin 2a receptor. Nonetheless, there is a wide variety in the subjective effects they can elicit (85), which is attributed to, among others, the binding affinities for serotonin receptor subtypes. Stimulants, on the other hand, typically act on multiple neurotransmitter systems, such as dopamine, noradrenaline, and serotonin (86, 87). However, based on their binding profiles, it is difficult to predict and compare effects (88). Therefore, to make a fair judgment on the neurocognitive and psychotomimetic effects and addictive potential of different drugs, it is advised to compare them based on psychotropic dose equivalence. When using psychotropic dose equivalence in other drug classes, it is advised to use the primary subjective motive for that class of drugs, e.g., feeling energetic with stimulants, or experiencing broadened consciousness with psychedelics (89). In the current study, subjective intoxication levels were used to determine psychotropic dose equivalence, as feeling high is the primary goal for recreational users of cannabinoids.

Up until now, more than 190 SCs have been reported by the EMCDDA, and only for a few of them the acute effects have been studied in humans (29–32, 90, 91). In addition, the most recent generations of SCs, which show increased potency and efficacy at CB1 receptors (92, 93), have not yet been tested in controlled studies. Whether using equipotent dose or psychotropic dose equivalence, controlled clinical studies with SCs and other NPS are challenging for ethical and safety reasons. A safe starting dose should be determined using all available preclinical data on pharmacology, toxicology, pharmacokinetics, and pharmacodynamics, including information retrieved from recreational users. It is vital to find a balance between minimizing the risks and maximizing the benefits (i.e., eliciting a pharmacological response). Furthermore, first-in-human studies with NPS should be conducted as phase 1 studies and implement the guidelines and regulations associated with this type of research (94).

## Conclusions

Findings in the present study suggest that the method of psychotropic dose equivalence is more accurate to predict drug outcome as compared to the method of dose equivalence (i.e., based on differences in potency). At psychotropic dose equivalence, THC and JWH-018 impaired cognitive performance to a similar extent, while dissociative effects were more pronounced for JWH-018 than for THC, and positive mood states were more affected by THC. The effects of JWH-018 that we reported in previous laboratory studies may have underestimated the neurocognitive and psychotomimetic effects that SC users may experience in real life when they achieve their maximal desired state of subjective high. In addition, with the high potency of SCs and the inconsistent content of smoking mixtures, it is very difficult for users to predict the maximal subjective high, resulting in very unpredictable neurocognitive outcomes and common overdosing.

## Data Availability Statement

The original contributions presented in the study are included in the article/Supplementary Material, further inquiries can be directed to the corresponding author.

## Ethics Statement

The studies involving human participants were reviewed and approved by Medisch-Ethische Toetsingscommissie azM/UM. The patients/participants provided their written informed consent to participate in this study.

## Author Contributions

ET: analysis of data and writing manuscript. JR, NM, and ET: interpretation of data. All authors: conceptualization and reviewing the manuscript. All authors approved the submitted version of the manuscript and agreed to be accountable for all aspects of the work.

## Conflict of Interest

The authors declare that the research was conducted in the absence of any commercial or financial relationships that could be construed as a potential conflict of interest.

## Publisher's Note

All claims expressed in this article are solely those of the authors and do not necessarily represent those of their affiliated organizations, or those of the publisher, the editors and the reviewers. Any product that may be evaluated in this article, or claim that may be made by its manufacturer, is not guaranteed or endorsed by the publisher.
