# Supplementary material for: A Comparison of Acute Neurocognitive and Psychotomimetic Effects of a Synthetic Cannabinoid and Natural Cannabis at Psychotropic Dose Equivalence
Source: Front Psychiatry. 2022 May 19;13:891811. doi: 10.3389/fpsyt.2022.891811 (PMC9160432; doi:10.3389/fpsyt.2022.891811)
Supplement: Supplementary file 1 [file Table_1.DOCX]

**Supplementary material**

Table 2. Means (SE) and *F*- and p-values for Drug, Study and Drug x Study effects in cognitive tasks with normally distributed data

|  | THC study | | JWH-018 study | | Drug effect | | Study | | Drug x study | |
| --- | --- | --- | --- | --- | --- | --- | --- | --- | --- | --- |
|  | Placebo | THC | Placebo | JWH-018 | *F* | p | *F* | p | *F* | p |
| CTT lambda-c (rad/sec) | 3.6 (.12) | 3.48 (.13) | 3.25 (.13) | 2.87 (.13) | 9.85 | **.003** | 8.88 | .005 | 2.89 | .096 |
| DAT tracking error (mm) | 17.04 (1.05) | 19.8 (.53) | 19.07 (.94) | 21.36 (.61) | 17.66 | **.00** | 3.35 | .074 | .16 | .69 |
| DAT RT (msec) | 2052.21 (57.92) | 2094.33 (66.22) | 2038.92 (55.69) | 2186.21 (60.19) | 5.25 | **.027** | .279 | .600 | 1.62 | .210 |
| MFFT latency (msec) | 9751.41 (588.08) | 11478.34 (830.28) | 13770.82 (1037.44) | 14310.98 (1682.03) | 1.067 | .307 | 9.274 | .004 | .292 | .591 |
| MFFT I-score | .06 (.32) | -.05 (.33) | -.18 (.24) | .17 (.36) | .248 | .621 | .000 | .998 | .905 | .347 |

Table 3. Overview of the mean (SEM) scores on the non-normally distributed cognitive variables and result of the Wilcoxon signed-rank test, to test for significant differences between drug condition and placebo, and mean (SEM) difference scores (THC or JWH-018 – placebo), and the results of the Mann-Whitney test, to assess for differences between THC and JWH-018.

|  | **Mean (SEM)** | | Wilxocon signed-rank test | | | **Mean (SEM) Difference scores** | | **Mann-Whitney test** | | | |
| --- | --- | --- | --- | --- | --- | --- | --- | --- | --- | --- | --- |
|  | Placebo | Drug | Z | p | r | THC | JWH-018 | Median THC (mean rank) | Median JWH-018 (mean rank) | U | p |
| DAT control losses (#) | 9.13 (1.6) | 22.58 (3.57) | -4.83 | **<.01** | .49 | 6.69 (2.1) | 20.22 (5.0) | 9.5 (20.7) | 19.5 (28.3) | 197 | .06 |
| DAT false alarms (#) | 3.02 (.34) | 6.02 (.77) | -4.14 | **<.01** | -.42 | 2.79 (.93) | 3.21 (1.41) | 5.0 (24.2) | 5 (24.8) | 281 | .89 |
| SST stop RT (msec) | 293.02 (10.8) | 302.38 (10.44) | -.81 | .418 |  | 19.17 (17.08) | -.46 (14.9) | 283.5 (28.1) | 288.0 (23.9) | 274 | .77 |
| SST Go RT (msec) | 691.85 (23.58) | 671.02 (24.63) | -1.14 | .255 |  | -43.93 (24.29) | 2.24 (16.3) | 585.11 (22.0) | 680.87 (27.0) | 229 | .22 |
| SST omission errors (#) | 1.52 (.47) | 3.73 (1.04) | -2.50 | **.012** | -.26 | .75 (.46) | 3.67 (1.73) | 1.0 (23.9) | 2.0 (25.1) | 274.5 | .78 |
| SST commission errors (#) | 9.33 (1.17) | 12.52 (1.5) | -2.68 | **<.01** | -.27 | 5.58 (1.88) | .79 (.72) | 13.5 (27.7) | 7.5 (21.3) | 211 | .11 |
| MFFT errors (#) | 3.75 (.59) | 5.4 (.64) | -1.77 | .077 |  | 1.08 (.66) | 2.21(1.57) | 4.0 (24.2) | 5.5 (24.8) | 280 | .87 |
| MFFT E-score | 1.34 (.16) | .66 (.19) | -2.17 | **.030** |  | -.85 (.27) | -.50 (.47) | .87 (22.9) | .85 (26.1) | 250 | .43 |

Table 4. Overview of the mean (SEM) scores on the subjective questionnaires and result of the Wilcoxon signed-rank test, to test for significant differences between drug condition and placebo, and mean (SEM) difference scores (THC or JWH-018 – placebo), and the results of the Mann-Whitney test, to assess for differences between THC and JWH-018.

|  | **Mean (SEM)** | | **Wilcoxon** signed-rank test | | | **Mean (SEM) Difference scores** | | **Mann-Whitney test** | | | | |
| --- | --- | --- | --- | --- | --- | --- | --- | --- | --- | --- | --- | --- |
|  | Placebo | Drug | Z | p | r | THC | JWH-018 | Median THC (mean rank) | Median JWH-018 (mean rank) | U | p | r |
| **POMS** |  | | | | | | | | | | | |
| Anxiety | 4.0 (.59) | 6.56 (.75) | -3.26 | **<.01** | -.33 | 4.25 (1.31) | .88 (1.1) | 2.5 (27.2) | 2.0 (21.8) | 224.5 | .19 |  |
| Depression | 1.73 (.50 | 3.48 (.89) | -2.21 | **.027** | -.23 | 3.7 (1.41) | -.21 (1.1) | .00 (27.8) | .00 (21.2) | 208 | .09 |  |
| Anger | 1.88 (.38) | 2.94 (.58) | -1.36 | .17 |  | .58 (.79) | 1.54 (.92) | .00 (22.2) | 1.0 (26.8) | 232 | .24 |  |
| Vigor | 10.17 (.88) | 8.15 (.87) | -2.62 | **<.01** | -.27 | -4.29 (.79) | .25 (1.2) | -3.5 (18.4) | 1.0 (30.6) | 141 | **<.01** | -.31 |
| Fatigue | 2.98 (.56) | 6.69 (.78) | -5.03 | **<.01** | -.51 | 4.08 (.84) | 3.33 (.99) | 3.0 (26.3) | 2.5 (22.7) | 246 | .38 |  |
| Confusion | 5.10 (.43) | 9.17 (.59) | -5.32 | **<.01** | -.54 | .43 (.85) | 3.79 (.80) | 3.5 (25.3) | 3.0 (23.7) | 269.5 | .70 |  |
| Friendliness | 16.35 (.90) | 13.73 (1.01) | -3.00 | **<.01** | -.31 | -5.04 (.87) | -.21 (1.17) | -5.0 (18.7) | -.5 (30.3) | 148 | **<.01** | -.29 |
| Elation | 8.67 (.69)  6 | 7.65 (.69) | -2.11 | **<.01** | -.22 | -2.75 (.62) | .71 (.98) | -2.5 (19.2) | .0 (29.8) | 161 | **<.01** | -.27 |
| Arousal | 6.08 (1.29) | -1.15 (.69) | -4.63 | **<.01** | -.47 | -8.46 (1.6) | -6.0 (2.19) | -6.5 (21.3) | -4.5 (27.7) | 211.5 | .11 |  |
| Positive mood | 6.94 (1.0) | 4.25 (1.24) | -2.45 | **.014** | -.25 | -6.25 (1.61) | .88 (1.8) | -3.5 (19.6) | -.5 (29.4) | 169.5 | **.014** | -.25 |
| **Bowdle** | | | | | | | | | | | | |
| Bowdle Externalization | .34(.01) | .55(.02 | -5.58 | **<.01** | -.22 | .2 (.04) | .21 (.04) | .20 (23.8) | .21 (25.2) | 271.5 | .73 |  |
| Bowdle Internalization | .32(.01) | .44(.02 | -5.14 | **<.01** | -.57 | .1 (.03) | .13 (.03) | .04 (21.8) | .09 (27.2) | 222 | .17 |  |
| Bowdle High | .28(.08) | 5.11(0.38) | -5.78 | **<.01** | -.52 | 5.06 (.56) | 4.61 (.54) | 5.0 (25.5) | 5.15 (23.5) | 263 | .61 |  |
| Bowdle Drowsy | .88(.24) | 3.17(.41) | -4.36 | **<.01** | -.59 | 1.79 (.58) | 2.78 (.65) | 1.50 (21.7) | 1.80 (27.3) | 221 | .16 |  |
| CADSS | | | | | | | | | | | | |
| CADSS depersonalisation | .40(.19) | 2.98(.59) | -5.58 | **<.01** | -.42 | .63 (.47) | 4.54 (1.01) | .0 (18.9) | 2.5 (30.1) | 153.5 | **<.01** | -.29 |
| Cadss derealisation | .81(.22) | 9.38(1.25) | -5.14 | **<.01** | -.57 | 4.25 (.82) | 12.88 (2.03) | 4.5 (17.8) | 11.0 (31.2) | 128 | **<.01** | -.34 |
| Cadds Amnesia | .21(.09) | 1.31(.24) | -5.78 | **<.01** | -.40 | .33 (.23) | 1.88 (.4) | .0 (18.7) | 1.0 (30.3) | 149.5 | **<.01** | -.31 |
| Cadss total | 1.42(.45) | 13.67(1.96) | -4.36 | **<.01** | -.55 | 5.21 (1.29) | 19.29 (3.18) | .0 (17.6) | .5 (31.4) | 123.5 | **<.01** | -.35 |
